# Audiovisual integration: an introduction to behavioral and neuro-cognitive methods

**DOI:** 10.3389/fpsyg.2013.00642

**Published:** 2013-09-17

**Authors:** Nicholas Altieri

**Affiliations:** Communication Sciences and Disorders, Idaho State UniversityPocatello, ID, USA

**Keywords:** audiovisual speech, integration, brain, speech and cognition, neuroimaging of speech, quantitative methods multisensory speech

Advances in neurocognitive and quantitative behavioral techniques have offered new insights to the study of cognition and language perception. This includes ways in which neurological processes and behavior are intimately intertwined. Examining traditional behavioral measures and model predictions, along with neurocognitive measures, will provide a powerful theory-driven and unified approach for researchers in the cognitive and language sciences. In this topic, the aim was to highlight some of the noteworthy methodological developments in the burgeoning field of multisensory speech perception.

Decades of research on audiovisual speech integration has, broadly speaking, reshaped the way language processing is conceptualized in the field. Beginning with Sumby and Pollack's seminal study of audiovisual integration published in 1954, qualitative and quantitative relationships have emerged showing the benefit of being able to obtain visual cues from “speech reading” under noisy conditions. A pioneering study by McGurk and MacDonald (1976) further demonstrated a form of integration phenomenon in which incongruent auditory-visual speech signals contribute to a fused or combined percept. (One such example is an auditory “ba” dubbed over a video of a talker articulating the syllable “ga.” This often yields a combined percept of “da.”)

Methods for determining whether “integration” occurs have, for example, involved examining whether a listener is susceptible to the McGurk effect, as we shall in a study by Setti et al. (2013) in the Research Topic. Perhaps a more commonly used assessment tool for determining the presence of “integration” has been measuring the extent to which a dependent variable (accuracy, speed, etc.) obtained from audiovisual trials is significantly “better” than the predicted response obtained from the unisensory conditions. A difference between obtained and predicted measures is thought to indicate a violation of independence between modalities (Altieri and Townsend, 2011; Altieri et al., 2013). In recent years, the neurological bases of these multisensory phenomena in speech perception have been developed largely in parallel with advances in behavioral techniques. Neuroimaging studies have looked at the Blood Oxygen-Level Dependent (BOLD) signal in relation to AV speech stimuli and compared that to the unisensory BOLD responses (e.g., Calvert, 2001; Stevenson and James, 2009). Within the milieu of EEG studies, similar comparisons have been made between the amplitude evoked by audiovisual, vs. auditory and visual-only stimuli. Similar to the fMRI studies, EEG research has contributed to the idea that integration occurs if the AV response differs from the unisensory responses (AV_ERP_ < A_ERP_ + V_ERP_; see, van Wassenhove et al., 2005; and Winneke and Phillips, 2011).

The application of EEG, fMRI or other imaging techniques in combination with behavioral indexes has therefore enhanced the testability of neural based theories of multisensory language processing. The broader aim of this Research Topic was to investigate the variety of manners in which neural measures of multisensory language processing could be anchored to behavioral indices of integration.

Several pioneering studies appear in this volume addressing a wide variety of issues in multisensory speech recognition. Quite significantly, this research explores integration in different age groups, for individuals with sensory processing deficits, and across different listening environments. First, a study carried out by Altieri and Wenger (2013) sought to rigorously associate the dynamic psychophysical measures of perception—namely the reaction time measure of *workload capacity* (Townsend and Nozawa, 1995)—with a neural dynamics from EEG. Under degraded listening conditions, we observed an increase in integration efficiency as measured by capacity, which co-occurred with an increase in multisensory ERPs relative to auditory-only ERPs. In a much needed review on the rules giving rise to multisensory integration, van Wassenhove (2013) provided an overview of “predictive coding hypotheses.” Updated hypotheses were considered, namely concerning how internal predictions about linguistics percepts are formulated. An overview of neuroimaging literature was included in the discussion.

Three reports explored the temporal effects of visual information on auditory encoding. One, provided by Ten Oever et al. (2013), varied the synchrony of the auditory and visual signals to explore the temporal effects of auditory syllable encoding. The results indicated a larger time-window for congruent AV syllables. Second, Moradi et al. (2013) provided a report investigating the influence of visual information on temporal recognition. This study showed that visual cues sped-up linguistic recognition in both noisy and clear listening conditions. Finally, a review and hypothesis article by Hertrich et al. (2013) proposes a brain network explaining how blind individuals, on average, are capable of perceiving auditory speech at a much faster rate compared to individuals with normal vision. Together, these articles will help constrain dynamic and neural-based theories regarding temporal aspects of audiovisual speech perception.

Two studies in this Research Topic also explored the effects of aging and neural development on perceptual skills. Kushnerenko et al. (2013) used an eye tracking paradigm in conjunction with ERPs to investigate the extent to which these measures predict normal linguistic development in children. Second, Setti et al. (2013) investigated integration skills by looking at whether age is predictive of the susceptibility to the McGurk effect. Interestingly, the authors found that older adults were more susceptible to the fusion than younger ones—ostensibly due to differences in perceptual rather than higher order cognitive processing abilities.

These research and review articles provide a rich introduction to a variety of fascinating techniques for investigating speech integration. Ideally, these research directions will pave the way toward a much improved tapestry of methodologies, and refinements of neuro-cognitive theories of multisensory processing across life-span, listening conditions, and sensory-cognitive abilities.
